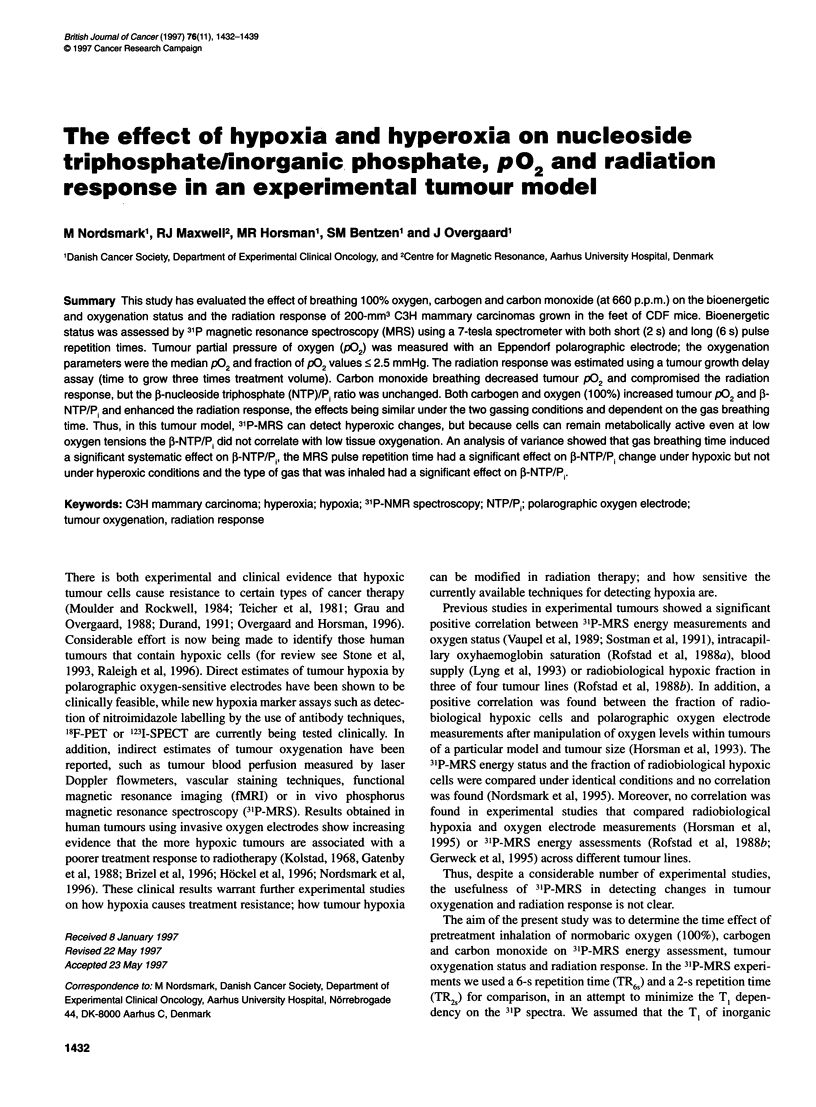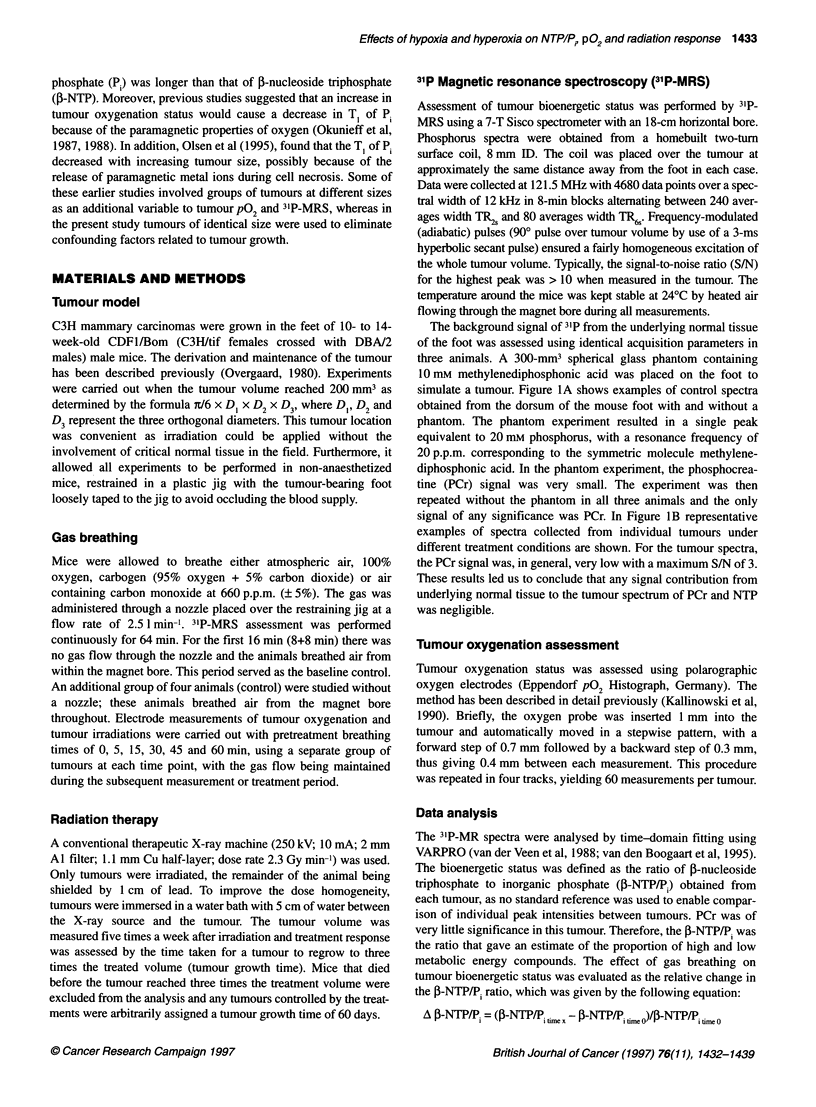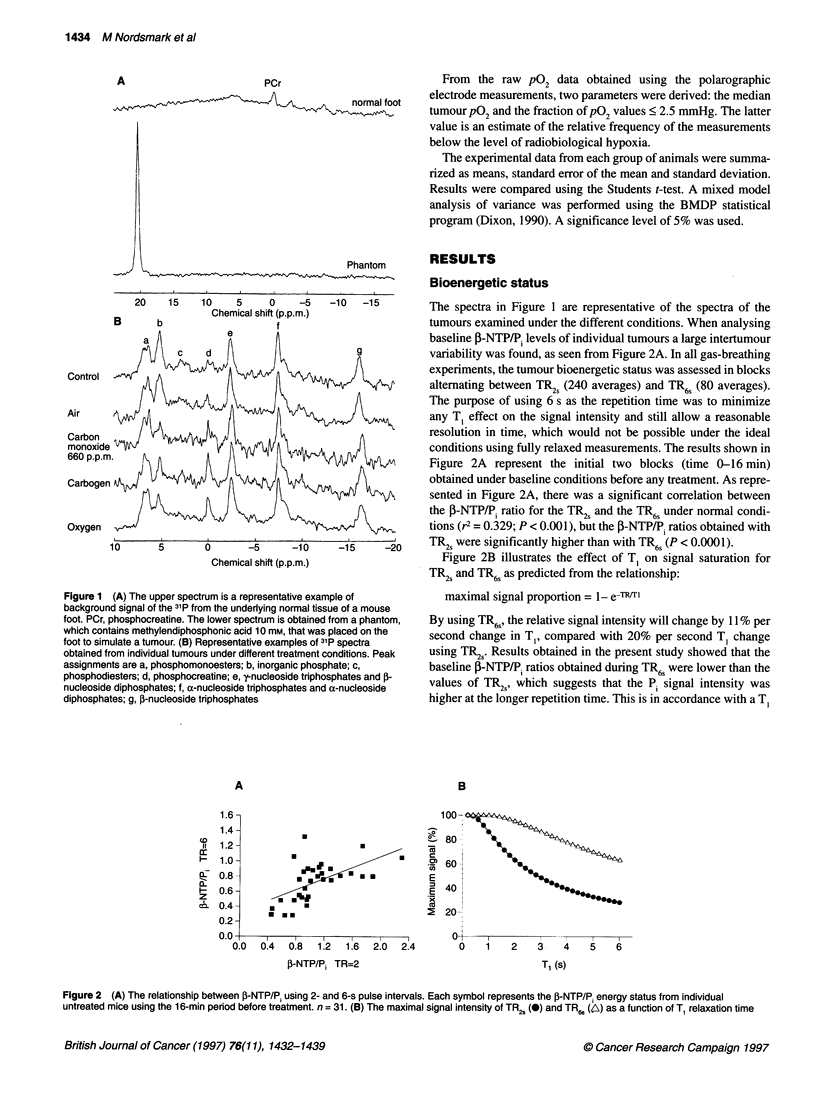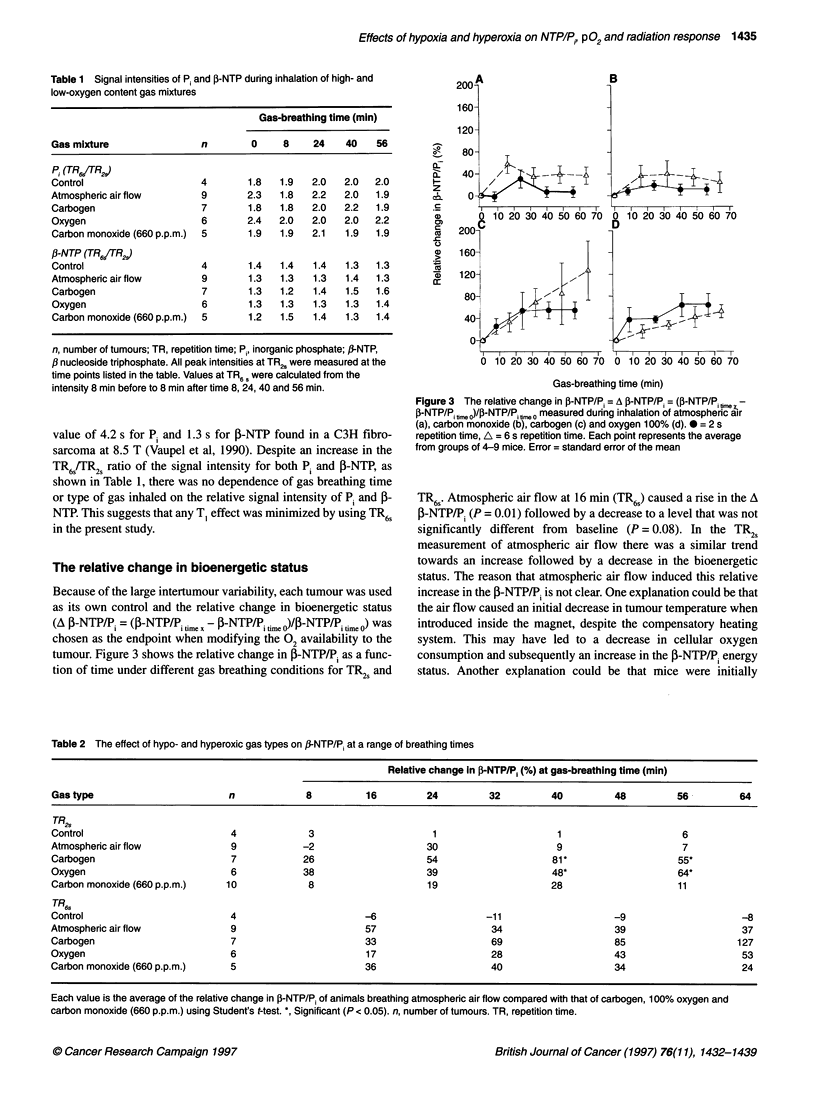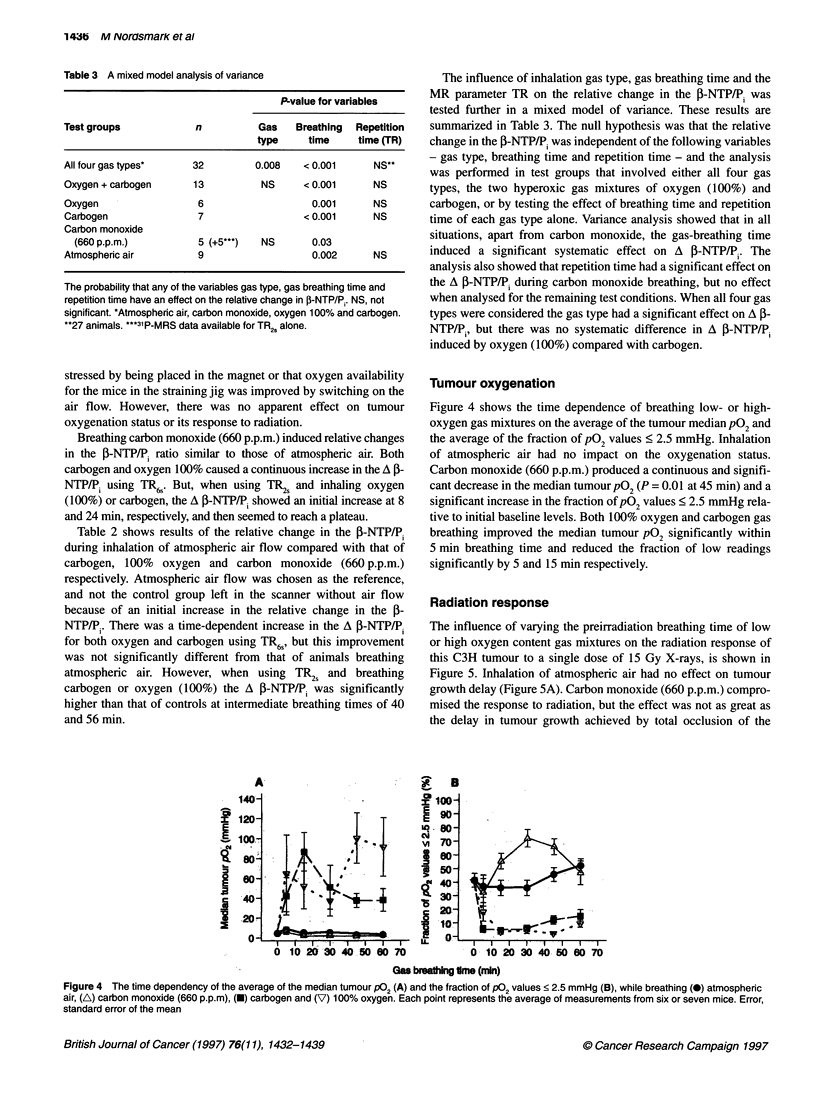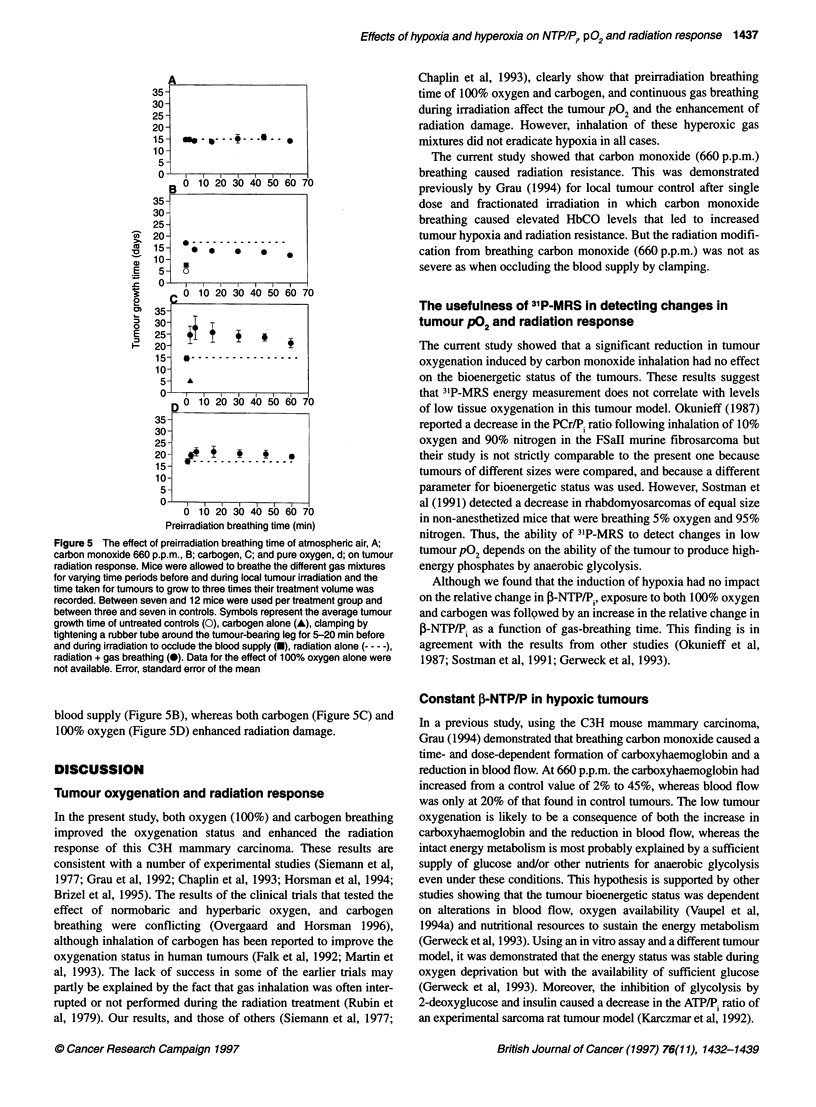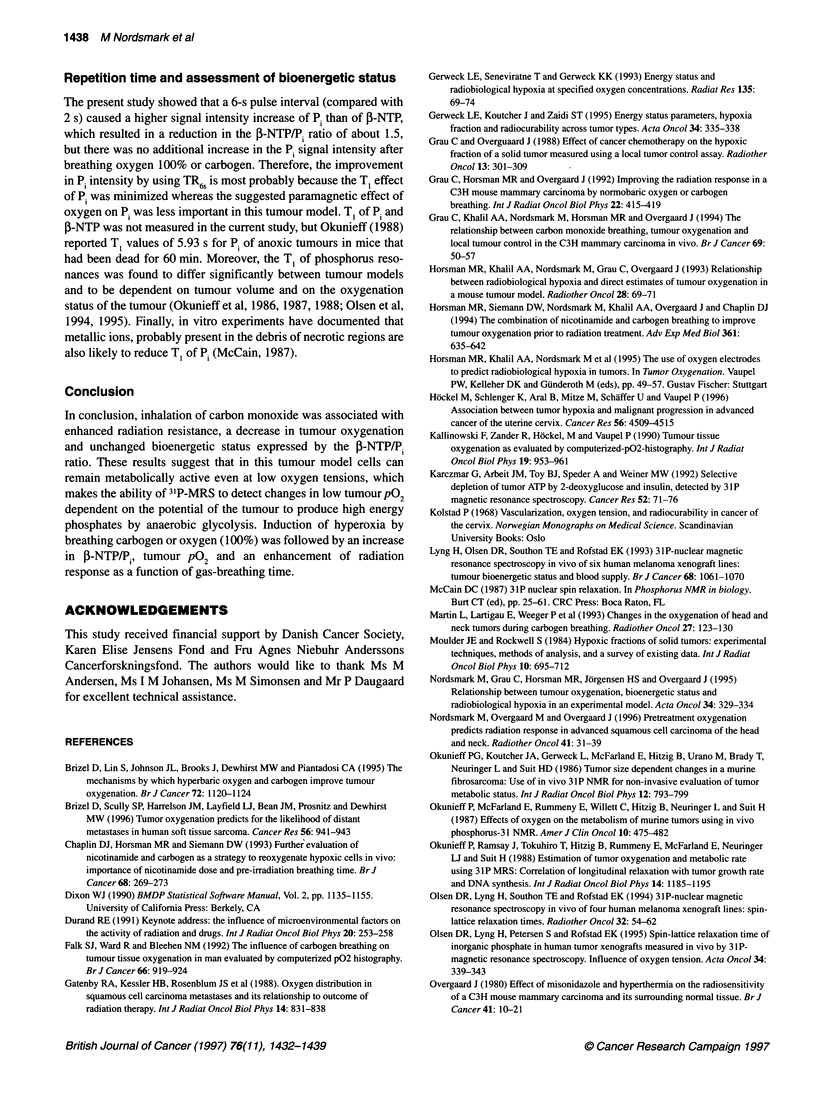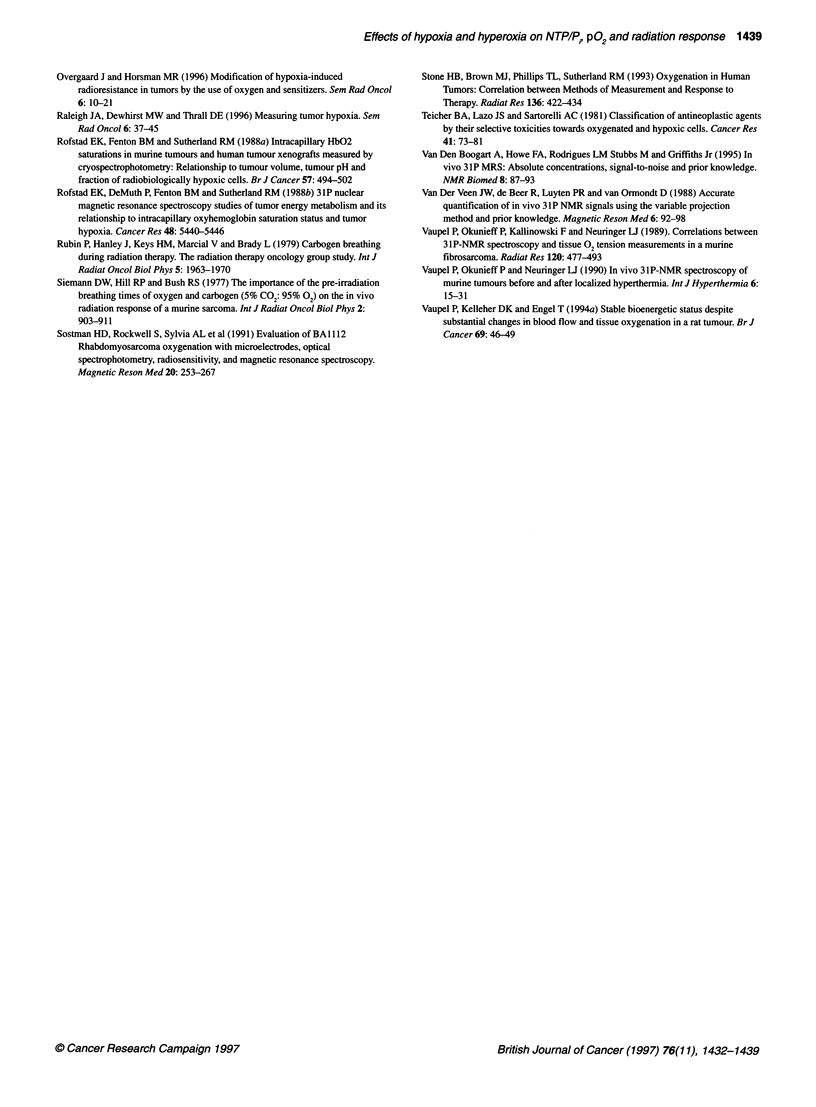# The effect of hypoxia and hyperoxia on nucleoside triphosphate/inorganic phosphate, pO2 and radiation response in an experimental tumour model.

**DOI:** 10.1038/bjc.1997.575

**Published:** 1997

**Authors:** M. Nordsmark, R. J. Maxwell, M. R. Horsman, S. M. Bentzen, J. Overgaard

**Affiliations:** Danish Cancer Society, Department of Experimental Clinical Oncology, Aarhus University Hospital, Aarhus C.

## Abstract

**Images:**


British Journal of Cancer (1997) 76(11), 1432–1439
© 1997 Cancer Research Campaign

### The effect of hypoxia and hyperoxia on nucleoside triphosphate/inorganic phosphate, $pO_2$ and radiation response in an experimental tumour model

**M Nordsmark[1], RJ Maxwell[2], MR Horsman[1], SM Bentzen[1] and J Overgaard[1]**

[1]Danish Cancer Society, Department of Experimental Clinical Oncology, and [2]Centre for Magnetic Resonance, Aarhus University Hospital, Denmark

**Summary** This study has evaluated the effect of breathing 100% oxygen, carbogen and carbon monoxide (at 660 p.p.m.) on the bioenergetic and oxygenation status and the radiation response of 200-mm³ C3H mammary carcinomas grown in the feet of CDF mice. Bioenergetic status was assessed by $^{31}P$ magnetic resonance spectroscopy (MRS) using a 7-tesla spectrometer with both short (2 s) and long (6 s) pulse repetition times. Tumour partial pressure of oxygen ($pO_2$) was measured with an Eppendorf polarographic electrode; the oxygenation parameters were the median $pO_2$ and fraction of $pO_2$ values ≤ 2.5 mmHg. The radiation response was estimated using a tumour growth delay assay (time to grow three times treatment volume). Carbon monoxide breathing decreased tumour $pO_2$ and compromised the radiation response, but the β-nucleoside triphosphate (NTP)/$P_i$ ratio was unchanged. Both carbogen and oxygen (100%) increased tumour $pO_2$ and β-NTP/$P_i$ and enhanced the radiation response, the effects being similar under the two gassing conditions and dependent on the gas breathing time. Thus, in this tumour model, $^{31}P$-MRS can detect hyperoxic changes, but because cells can remain metabolically active even at low oxygen tensions the β-NTP/$P_i$ did not correlate with low tissue oxygenation. An analysis of variance showed that gas breathing time induced a significant systematic effect on β-NTP/$P_i$, the MRS pulse repetition time had a significant effect on β-NTP/$P_i$ change under hypoxic but not under hyperoxic conditions and the type of gas that was inhaled had a significant effect on β-NTP/$P_i$.

**Keywords:** C3H mammary carcinoma; hyperoxia; hypoxia; $^{31}P$-NMR spectroscopy; NTP/$P_i$; polarographic oxygen electrode; tumour oxygenation, radiation response

There is both experimental and clinical evidence that hypoxic tumour cells cause resistance to certain types of cancer therapy (Moulder and Rockwell, 1984; Teicher et al, 1981; Grau and Overgaard, 1988; Durand, 1991; Overgaard and Horsman, 1996). Considerable effort is now being made to identify those human tumours that contain hypoxic cells (for review see Stone et al, 1993, Raleigh et al, 1996). Direct estimates of tumour hypoxia by polarographic oxygen-sensitive electrodes have been shown to be clinically feasible, while new hypoxia marker assays such as detection of nitroimidazole labelling by the use of antibody techniques, $^{18}F$-PET or $^{123}I$-SPECT are currently being tested clinically. In addition, indirect estimates of tumour oxygenation have been reported, such as tumour blood perfusion measured by laser Doppler flowmeters, vascular staining techniques, functional magnetic resonance imaging (fMRI) or in vivo phosphorus magnetic resonance spectroscopy ($^{31}P$-MRS). Results obtained in human tumours using invasive oxygen electrodes show increasing evidence that the more hypoxic tumours are associated with a poorer treatment response to radiotherapy (Kolstad, 1968, Gatenby et al, 1988; Brizel et al, 1996; Höckel et al, 1996; Nordsmark et al, 1996). These clinical results warrant further experimental studies on how hypoxia causes treatment resistance; how tumour hypoxia can be modified in radiation therapy; and how sensitive the currently available techniques for detecting hypoxia are.

Previous studies in experimental tumours showed a significant positive correlation between $^{31}P$-MRS energy measurements and oxygen status (Vaupel et al, 1989; Sostman et al, 1991), intracapillary oxyhaemoglobin saturation (Rofstad et al, 1988*a*), blood supply (Lyng et al, 1993) or radiobiological hypoxic fraction in three of four tumour lines (Rofstad et al, 1988*b*). In addition, a positive correlation was found between the fraction of radiobiological hypoxic cells and polarographic oxygen electrode measurements after manipulation of oxygen levels within tumours of a particular model and tumour size (Horsman et al, 1993). The $^{31}P$-MRS energy status and the fraction of radiobiological hypoxic cells were compared under identical conditions and no correlation was found (Nordsmark et al, 1995). Moreover, no correlation was found in experimental studies that compared radiobiological hypoxia and oxygen electrode measurements (Horsman et al, 1995) or $^{31}P$-MRS energy assessments (Rofstad et al, 1988*b*; Gerweck et al, 1995) across different tumour lines.

Thus, despite a considerable number of experimental studies, the usefulness of $^{31}P$-MRS in detecting changes in tumour oxygenation and radiation response is not clear.

The aim of the present study was to determine the time effect of pretreatment inhalation of normobaric oxygen (100%), carbogen and carbon monoxide on $^{31}P$-MRS energy assessment, tumour oxygenation status and radiation response. In the $^{31}P$-MRS experiments we used a 6-s repetition time ($TR_{6s}$) and a 2-s repetition time ($TR_{2s}$) for comparison, in an attempt to minimize the $T_1$ dependency on the $^{31}P$ spectra. We assumed that the $T_1$ of inorganic

*Received 8 January 1997*
*Revised 22 May 1997*
*Accepted 23 May 1997*

*Correspondence to:* M Nordsmark, Danish Cancer Society, Department of Experimental Clinical Oncology, Aarhus University Hospital, Nörrebrogade 44, DK-8000 Aarhus C, Denmark phosphate ($P_i$) was longer than that of β-nucleoside triphosphate (β-NTP). Moreover, previous studies suggested that an increase in tumour oxygenation status would cause a decrease in $T_1$ of $P_i$ because of the paramagnetic properties of oxygen (Okunieff et al, 1987, 1988). In addition, Olsen et al (1995), found that the $T_1$ of $P_i$ decreased with increasing tumour size, possibly because of the release of paramagnetic metal ions during cell necrosis. Some of these earlier studies involved groups of tumours at different sizes as an additional variable to tumour $pO_2$ and $^{31}$P-MRS, whereas in the present study tumours of identical size were used to eliminate confounding factors related to tumour growth.

### MATERIALS AND METHODS

### Tumour model

C3H mammary carcinomas were grown in the feet of 10- to 14-week-old CDF1/Bom (C3H/tif females crossed with DBA/2 males) male mice. The derivation and maintenance of the tumour has been described previously (Overgaard, 1980). Experiments were carried out when the tumour volume reached 200 mm³ as determined by the formula $\pi/6 \times D_1 \times D_2 \times D_3$, where $D_1$, $D_2$ and $D_3$ represent the three orthogonal diameters. This tumour location was convenient as irradiation could be applied without the involvement of critical normal tissue in the field. Furthermore, it allowed all experiments to be performed in non-anaesthetized mice, restrained in a plastic jig with the tumour-bearing foot loosely taped to the jig to avoid occluding the blood supply.

### Gas breathing

Mice were allowed to breathe either atmospheric air, 100% oxygen, carbogen (95% oxygen + 5% carbon dioxide) or air containing carbon monoxide at 660 p.p.m. (± 5%). The gas was administered through a nozzle placed over the restraining jig at a flow rate of 2.5 l min⁻¹. $^{31}$P-MRS assessment was performed continuously for 64 min. For the first 16 min (8+8 min) there was no gas flow through the nozzle and the animals breathed air from within the magnet bore. This period served as the baseline control. An additional group of four animals (control) were studied without a nozzle; these animals breathed air from the magnet bore throughout. Electrode measurements of tumour oxygenation and tumour irradiations were carried out with pretreatment breathing times of 0, 5, 15, 30, 45 and 60 min, using a separate group of tumours at each time point, with the gas flow being maintained during the subsequent measurement or treatment period.

### Radiation therapy

A conventional therapeutic X-ray machine (250 kV; 10 mA; 2 mm Al filter; 1.1 mm Cu half-layer; dose rate 2.3 Gy min⁻¹) was used. Only tumours were irradiated, the remainder of the animal being shielded by 1 cm of lead. To improve the dose homogeneity, tumours were immersed in a water bath with 5 cm of water between the X-ray source and the tumour. The tumour volume was measured five times a week after irradiation and treatment response was assessed by the time taken for a tumour to regrow to three times the treated volume (tumour growth time). Mice that died before the tumour reached three times the treatment volume were excluded from the analysis and any tumours controlled by the treatments were arbitrarily assigned a tumour growth time of 60 days.

### $^{31}$P Magnetic resonance spectroscopy ($^{31}$P-MRS)

Assessment of tumour bioenergetic status was performed by $^{31}$P-MRS using a 7-T Sisco spectrometer with an 18-cm horizontal bore. Phosphorus spectra were obtained from a homebuilt two-turn surface coil, 8 mm ID. The coil was placed over the tumour at approximately the same distance away from the foot in each case. Data were collected at 121.5 MHz with 4680 data points over a spectral width of 12 kHz in 8-min blocks alternating between 240 averages width $TR_{2s}$ and 80 averages width $TR_{6s}$. Frequency-modulated (adiabatic) pulses (90° pulse over tumour volume by use of a 3-ms hyperbolic secant pulse) ensured a fairly homogeneous excitation of the whole tumour volume. Typically, the signal-to-noise ratio (S/N) for the highest peak was > 10 when measured in the tumour. The temperature around the mice was kept stable at 24°C by heated air flowing through the magnet bore during all measurements.

The background signal of $^{31}$P from the underlying normal tissue of the foot was assessed using identical acquisition parameters in three animals. A 300-mm³ spherical glass phantom containing 10 mM methylenediphosphonic acid was placed on the foot to simulate a tumour. Figure 1A shows examples of control spectra obtained from the dorsum of the mouse foot with and without a phantom. The phantom experiment resulted in a single peak equivalent to 20 mM phosphorus, with a resonance frequency of 20 p.p.m. corresponding to the symmetric molecule methylenediphosphonic acid. In the phantom experiment, the phosphocreatine (PCr) signal was very small. The experiment was then repeated without the phantom in all three animals and the only signal of any significance was PCr. In Figure 1B representative examples of spectra collected from individual tumours under different treatment conditions are shown. For the tumour spectra, the PCr signal was, in general, very low with a maximum S/N of 3. These results led us to conclude that any signal contribution from underlying normal tissue to the tumour spectrum of PCr and NTP was negligible.

### Tumour oxygenation assessment

Tumour oxygenation status was assessed using polarographic oxygen electrodes (Eppendorf $pO_2$ Histograph, Germany). The method has been described in detail previously (Kallinowski et al, 1990). Briefly, the oxygen probe was inserted 1 mm into the tumour and automatically moved in a stepwise pattern, with a forward step of 0.7 mm followed by a backward step of 0.3 mm, thus giving 0.4 mm between each measurement. This procedure was repeated in four tracks, yielding 60 measurements per tumour.

### Data analysis

The $^{31}$P-MR spectra were analysed by time–domain fitting using VARPRO (van der Veen et al, 1988; van den Boogaart et al, 1995). The bioenergetic status was defined as the ratio of β-nucleoside triphosphate to inorganic phosphate (β-NTP/$P_i$) obtained from each tumour, as no standard reference was used to enable comparison of individual peak intensities between tumours. PCr was of very little significance in this tumour. Therefore, the β-NTP/$P_i$ was the ratio that gave an estimate of the proportion of high and low metabolic energy compounds. The effect of gas breathing on tumour bioenergetic status was evaluated as the relative change in the β-NTP/$P_i$ ratio, which was given by the following equation:

$$\Delta\,\beta\text{-NTP/P}_i = (\beta\text{-NTP/P}_{i\,\text{time}\,x} - \beta\text{-NTP/P}_{i\,\text{time}\,0})/\beta\text{-NTP/P}_{i\,\text{time}\,0}$$

© Cancer Research Campaign 1997

**Figure 1** (**A**) The upper spectrum is a representative example of background signal of the $^{31}P$ from the underlying normal tissue of a mouse foot. PCr, phosphocreatine. The lower spectrum is obtained from a phantom, which contains methylendiphosphonic acid 10 mM, that was placed on the foot to simulate a tumour. (**B**) Representative examples of $^{31}P$ spectra obtained from individual tumours under different treatment conditions. Peak assignments are a, phosphomonoesters; b, inorganic phosphate; c, phosphodiesters; d, phosphocreatine; e, γ-nucleoside triphosphates and β-nucleoside diphosphates; f, α-nucleoside triphosphates and α-nucleoside diphosphates; g, β-nucleoside triphosphates

From the raw $pO_2$ data obtained using the polarographic electrode measurements, two parameters were derived: the median tumour $pO_2$ and the fraction of $pO_2$ values ≤ 2.5 mmHg. The latter value is an estimate of the relative frequency of the measurements below the level of radiobiological hypoxia.

The experimental data from each group of animals were summarized as means, standard error of the mean and standard deviation. Results were compared using the Students *t*-test. A mixed model analysis of variance was performed using the BMDP statistical program (Dixon, 1990). A significance level of 5% was used.

### RESULTS

### Bioenergetic status

The spectra in Figure 1 are representative of the spectra of the tumours examined under the different conditions. When analysing baseline β-NTP/$P_i$ levels of individual tumours a large intertumour variability was found, as seen from Figure 2A. In all gas-breathing experiments, the tumour bioenergetic status was assessed in blocks alternating between $TR_{2s}$ (240 averages) and $TR_{6s}$ (80 averages). The purpose of using 6 s as the repetition time was to minimize any $T_1$ effect on the signal intensity and still allow a reasonable resolution in time, which would not be possible under the ideal conditions using fully relaxed measurements. The results shown in Figure 2A represent the initial two blocks (time 0–16 min) obtained under baseline conditions before any treatment. As represented in Figure 2A, there was a significant correlation between the β-NTP/$P_i$ ratio for the $TR_{2s}$ and the $TR_{6s}$ under normal conditions ($r^2 = 0.329$; $P < 0.001$), but the β-NTP/$P_i$ ratios obtained with $TR_{2s}$ were significantly higher than with $TR_{6s}$ ($P < 0.0001$).

Figure 2B illustrates the effect of $T_1$ on signal saturation for $TR_{2s}$ and $TR_{6s}$ as predicted from the relationship:

$$\text{maximal signal proportion} = 1 - e^{-TR/T1}$$

By using $TR_{6s}$, the relative signal intensity will change by 11% per second change in $T_1$, compared with 20% per second $T_1$ change using $TR_{2s}$. Results obtained in the present study showed that the baseline β-NTP/$P_i$ ratios obtained during $TR_{6s}$ were lower than the values of $TR_{2s}$, which suggests that the $P_i$ signal intensity was higher at the longer repetition time. This is in accordance with a $T_1$

**Figure 2** (**A**) The relationship between β-NTP/$P_i$ using 2- and 6-s pulse intervals. Each symbol represents the β-NTP/$P_i$ energy status from individual untreated mice using the 16-min period before treatment. $n = 31$. (**B**) The maximal signal intensity of $TR_{2s}$ (●) and $TR_{6s}$ (△) as a function of $T_1$ relaxation time

© Cancer Research Campaign 1997

**Table 1** Signal intensities of $P_i$ and β-NTP during inhalation of high- and low-oxygen content gas mixtures

| Gas mixture | *n* | Gas-breathing time (min) | | | | |
|---|---|---|---|---|---|---|
| | | 0 | 8 | 24 | 40 | 56 |
| *$P_i$ ($TR_{6s}/TR_{2s}$)* | | | | | | |
| Control | 4 | 1.8 | 1.9 | 2.0 | 2.0 | 2.0 |
| Atmospheric air flow | 9 | 2.3 | 1.8 | 2.2 | 2.0 | 1.9 |
| Carbogen | 7 | 1.8 | 1.8 | 2.0 | 2.2 | 1.9 |
| Oxygen | 6 | 2.4 | 2.0 | 2.0 | 2.0 | 2.2 |
| Carbon monoxide (660 p.p.m.) | 5 | 1.9 | 1.9 | 2.1 | 1.9 | 1.9 |
| *β-NTP ($TR_{6s}/TR_{2s}$)* | | | | | | |
| Control | 4 | 1.4 | 1.4 | 1.4 | 1.3 | 1.3 |
| Atmospheric air flow | 9 | 1.3 | 1.3 | 1.3 | 1.4 | 1.3 |
| Carbogen | 7 | 1.3 | 1.2 | 1.4 | 1.5 | 1.6 |
| Oxygen | 6 | 1.3 | 1.3 | 1.3 | 1.3 | 1.4 |
| Carbon monoxide (660 p.p.m.) | 5 | 1.2 | 1.5 | 1.4 | 1.3 | 1.4 |

*n*, number of tumours; TR, repetition time; $P_i$, inorganic phosphate; *β*-NTP, *β* nucleoside triphosphate. All peak intensities at $TR_{2s}$ were measured at the time points listed in the table. Values at $TR_{6\,s}$ were calculated from the intensity 8 min before to 8 min after time 8, 24, 40 and 56 min.

value of 4.2 s for $P_i$ and 1.3 s for β-NTP found in a C3H fibrosarcoma at 8.5 T (Vaupel et al, 1990). Despite an increase in the $TR_{6s}/TR_{2s}$ ratio of the signal intensity for both $P_i$ and β-NTP, as shown in Table 1, there was no dependence of gas breathing time or type of gas inhaled on the relative signal intensity of $P_i$ and β-NTP. This suggests that any $T_1$ effect was minimized by using $TR_{6s}$ in the present study.

### The relative change in bioenergetic status

Because of the large intertumour variability, each tumour was used as its own control and the relative change in bioenergetic status ($\Delta$ β-NTP/$P_i$ = (β-NTP/$P_{i\,time\,x}$ − β-NTP/$P_{i\,time\,0}$)/β-NTP/$P_{i\,time\,0}$) was chosen as the endpoint when modifying the $O_2$ availability to the tumour. Figure 3 shows the relative change in β-NTP/$P_i$ as a function of time under different gas breathing conditions for $TR_{2s}$ and

**Figure 3** The relative change in β-NTP/$P_i$ = $\Delta$ β-NTP/$P_i$ = (β-NTP/$P_{i\,time\,x}$ − β-NTP/$P_{i\,time\,0}$)/β-NTP/$P_{i\,time\,0}$ measured during inhalation of atmospheric air (a), carbon monoxide (b), carbogen (c) and oxygen 100% (d). ● = 2 s repetition time, △ = 6 s repetition time. Each point represents the average from groups of 4–9 mice. Error = standard error of the mean

$TR_{6s}$. Atmospheric air flow at 16 min ($TR_{6s}$) caused a rise in the $\Delta$ β-NTP/$P_i$ ($P$ = 0.01) followed by a decrease to a level that was not significantly different from baseline ($P$ = 0.08). In the $TR_{2s}$ measurement of atmospheric air flow there was a similar trend towards an increase followed by a decrease in the bioenergetic status. The reason that atmospheric air flow induced this relative increase in the β-NTP/$P_i$ is not clear. One explanation could be that the air flow caused an initial decrease in tumour temperature when introduced inside the magnet, despite the compensatory heating system. This may have led to a decrease in cellular oxygen consumption and subsequently an increase in the β-NTP/$P_i$ energy status. Another explanation could be that mice were initially

**Table 2** The effect of hypo- and hyperoxic gas types on *β*-NTP/$P_i$ at a range of breathing times

| Gas type | *n* | Relative change in β-NTP/$P_i$ (%) at gas-breathing time (min) | | | | | | | |
|---|---|---|---|---|---|---|---|---|---|
| | | 8 | 16 | 24 | 32 | 40 | 48 | 56 | 64 |
| *$TR_{2s}$* | | | | | | | | | |
| Control | 4 | 3 | | 1 | | 1 | | 6 | |
| Atmospheric air flow | 9 | −2 | | 30 | | 9 | | 7 | |
| Carbogen | 7 | 26 | | 54 | | 81* | | 55* | |
| Oxygen | 6 | 38 | | 39 | | 48* | | 64* | |
| Carbon monoxide (660 p.p.m.) | 10 | 8 | | 19 | | 28 | | 11 | |
| *$TR_{6s}$* | | | | | | | | | |
| Control | 4 | | −6 | | −11 | | −9 | | −8 |
| Atmospheric air flow | 9 | | 57 | | 34 | | 39 | | 37 |
| Carbogen | 7 | | 33 | | 69 | | 85 | | 127 |
| Oxygen | 6 | | 17 | | 28 | | 43 | | 53 |
| Carbon monoxide (660 p.p.m.) | 5 | | 36 | | 40 | | 34 | | 24 |

Each value is the average of the relative change in β-NTP/$P_i$ of animals breathing atmospheric air flow compared with that of carbogen, 100% oxygen and carbon monoxide (660 p.p.m.) using Student's *t*-test. *, Significant ($P$ < 0.05). *n*, number of tumours. TR, repetition time.

© Cancer Research Campaign 1997 

**Table 3** A mixed model analysis of variance

| Test groups | n | P-value for variables | | |
|---|---|---|---|---|
| | | Gas type | Breathing time | Repetition time (TR) |
| All four gas types* | 32 | 0.008 | < 0.001 | NS** |
| Oxygen + carbogen | 13 | NS | < 0.001 | NS |
| Oxygen | 6 | | 0.001 | NS |
| Carbogen | 7 | | < 0.001 | NS |
| Carbon monoxide (660 p.p.m.) | 5 (+5***) | NS | 0.03 | |
| Atmospheric air | 9 | | 0.002 | NS |

The probability that any of the variables gas type, gas breathing time and repetition time have an effect on the relative change in β-NTP/$P_i$. NS, not significant. *Atmospheric air, carbon monoxide, oxygen 100% and carbogen. **27 animals. ***$^{31}$P-MRS data available for $TR_{2s}$ alone.

stressed by being placed in the magnet or that oxygen availability for the mice in the straining jig was improved by switching on the air flow. However, there was no apparent effect on tumour oxygenation status or its response to radiation.

Breathing carbon monoxide (660 p.p.m.) induced relative changes in the β-NTP/$P_i$ ratio similar to those of atmospheric air. Both carbogen and oxygen 100% caused a continuous increase in the Δ β-NTP/$P_i$ using $TR_{6s}$. But, when using $TR_{2s}$ and inhaling oxygen (100%) or carbogen, the Δ β-NTP/$P_i$ showed an initial increase at 8 and 24 min, respectively, and then seemed to reach a plateau.

Table 2 shows results of the relative change in the β-NTP/$P_i$ during inhalation of atmospheric air flow compared with that of carbogen, 100% oxygen and carbon monoxide (660 p.p.m.) respectively. Atmospheric air flow was chosen as the reference, and not the control group left in the scanner without air flow because of an initial increase in the relative change in the β-NTP/$P_i$. There was a time-dependent increase in the Δ β-NTP/$P_i$ for both oxygen and carbogen using $TR_{6s}$, but this improvement was not significantly different from that of animals breathing atmospheric air. However, when using $TR_{2s}$ and breathing carbogen or oxygen (100%) the Δ β-NTP/$P_i$ was significantly higher than that of controls at intermediate breathing times of 40 and 56 min.

The influence of inhalation gas type, gas breathing time and the MR parameter TR on the relative change in the β-NTP/$P_i$ was tested further in a mixed model of variance. These results are summarized in Table 3. The null hypothesis was that the relative change in the β-NTP/$P_i$ was independent of the following variables – gas type, breathing time and repetition time – and the analysis was performed in test groups that involved either all four gas types, the two hyperoxic gas mixtures of oxygen (100%) and carbogen, or by testing the effect of breathing time and repetition time of each gas type alone. Variance analysis showed that in all situations, apart from carbon monoxide, the gas-breathing time induced a significant systematic effect on Δ β-NTP/$P_i$. The analysis also showed that repetition time had a significant effect on the Δ β-NTP/$P_i$ during carbon monoxide breathing, but no effect when analysed for the remaining test conditions. When all four gas types were considered the gas type had a significant effect on Δ β-NTP/$P_i$, but there was no systematic difference in Δ β-NTP/$P_i$ induced by oxygen (100%) compared with carbogen.

### Tumour oxygenation

Figure 4 shows the time dependence of breathing low- or high-oxygen gas mixtures on the average of the tumour median $pO_2$ and the average of the fraction of $pO_2$ values ≤ 2.5 mmHg. Inhalation of atmospheric air had no impact on the oxygenation status. Carbon monoxide (660 p.p.m.) produced a continuous and significant decrease in the median tumour $pO_2$ ($P = 0.01$ at 45 min) and a significant increase in the fraction of $pO_2$ values ≤ 2.5 mmHg relative to initial baseline levels. Both 100% oxygen and carbogen gas breathing improved the median tumour $pO_2$ significantly within 5 min breathing time and reduced the fraction of low readings significantly by 5 and 15 min respectively.

### Radiation response

The influence of varying the preirradiation breathing time of low or high oxygen content gas mixtures on the radiation response of this C3H tumour to a single dose of 15 Gy X-rays, is shown in Figure 5. Inhalation of atmospheric air had no effect on tumour growth delay (Figure 5A). Carbon monoxide (660 p.p.m.) compromised the response to radiation, but the effect was not as great as the delay in tumour growth achieved by total occlusion of the

**Figure 4** The time dependency of the average of the median tumour $pO_2$ (**A**) and the fraction of $pO_2$ values ≤ 2.5 mmHg (**B**), while breathing (●) atmospheric air, (△) carbon monoxide (660 p.p.m), (■) carbogen and (▽) 100% oxygen. Each point represents the average of measurements from six or seven mice. Error, standard error of the mean

© Cancer Research Campaign 1997

**Figure 5** The effect of preirradiation breathing time of atmospheric air, A; carbon monoxide 660 p.p.m., B; carbogen, C; and pure oxygen, d; on tumour radiation response. Mice were allowed to breathe the different gas mixtures for varying time periods before and during local tumour irradiation and the time taken for tumours to grow to three times their treatment volume was recorded. Between seven and 12 mice were used per treatment group and between three and seven in controls. Symbols represent the average tumour growth time of untreated controls (○), carbogen alone (▲), clamping by tightening a rubber tube around the tumour-bearing leg for 5–20 min before and during irradiation to occlude the blood supply (■), radiation alone (- - - -), radiation + gas breathing (●). Data for the effect of 100% oxygen alone were not available. Error, standard error of the mean blood supply (Figure 5B), whereas both carbogen (Figure 5C) and 100% oxygen (Figure 5D) enhanced radiation damage.

### DISCUSSION

### Tumour oxygenation and radiation response

In the present study, both oxygen (100%) and carbogen breathing improved the oxygenation status and enhanced the radiation response of this C3H mammary carcinoma. These results are consistent with a number of experimental studies (Siemann et al, 1977; Grau et al, 1992; Chaplin et al, 1993; Horsman et al, 1994; Brizel et al, 1995). The results of the clinical trials that tested the effect of normobaric and hyperbaric oxygen, and carbogen breathing were conflicting (Overgaard and Horsman 1996), although inhalation of carbogen has been reported to improve the oxygenation status in human tumours (Falk et al, 1992; Martin et al, 1993). The lack of success in some of the earlier trials may partly be explained by the fact that gas inhalation was often interrupted or not performed during the radiation treatment (Rubin et al, 1979). Our results, and those of others (Siemann et al, 1977; Chaplin et al, 1993), clearly show that preirradiation breathing time of 100% oxygen and carbogen, and continuous gas breathing during irradiation affect the tumour $pO_2$ and the enhancement of radiation damage. However, inhalation of these hyperoxic gas mixtures did not eradicate hypoxia in all cases.

The current study showed that carbon monoxide (660 p.p.m.) breathing caused radiation resistance. This was demonstrated previously by Grau (1994) for local tumour control after single dose and fractionated irradiation in which carbon monoxide breathing caused elevated HbCO levels that led to increased tumour hypoxia and radiation resistance. But the radiation modification from breathing carbon monoxide (660 p.p.m.) was not as severe as when occluding the blood supply by clamping.

### The usefulness of $^{31}$P-MRS in detecting changes in tumour $pO_2$ and radiation response

The current study showed that a significant reduction in tumour oxygenation induced by carbon monoxide inhalation had no effect on the bioenergetic status of the tumours. These results suggest that $^{31}$P-MRS energy measurement does not correlate with levels of low tissue oxygenation in this tumour model. Okunieff (1987) reported a decrease in the PCr/$P_i$ ratio following inhalation of 10% oxygen and 90% nitrogen in the FSaII murine fibrosarcoma but their study is not strictly comparable to the present one because tumours of different sizes were compared, and because a different parameter for bioenergetic status was used. However, Sostman et al (1991) detected a decrease in rhabdomyosarcomas of equal size in non-anesthetized mice that were breathing 5% oxygen and 95% nitrogen. Thus, the ability of $^{31}$P-MRS to detect changes in low tumour $pO_2$ depends on the ability of the tumour to produce high-energy phosphates by anaerobic glycolysis.

Although we found that the induction of hypoxia had no impact on the relative change in β-NTP/$P_i$, exposure to both 100% oxygen and carbogen was followed by an increase in the relative change in β-NTP/$P_i$ as a function of gas-breathing time. This finding is in agreement with the results from other studies (Okunieff et al, 1987; Sostman et al, 1991; Gerweck et al, 1993).

### Constant β-NTP/P in hypoxic tumours

In a previous study, using the C3H mouse mammary carcinoma, Grau (1994) demonstrated that breathing carbon monoxide caused a time- and dose-dependent formation of carboxyhaemoglobin and a reduction in blood flow. At 660 p.p.m. the carboxyhaemoglobin had increased from a control value of 2% to 45%, whereas blood flow was only at 20% of that found in control tumours. The low tumour oxygenation is likely to be a consequence of both the increase in carboxyhaemoglobin and the reduction in blood flow, whereas the intact energy metabolism is most probably explained by a sufficient supply of glucose and/or other nutrients for anaerobic glycolysis even under these conditions. This hypothesis is supported by other studies showing that the tumour bioenergetic status was dependent on alterations in blood flow, oxygen availability (Vaupel et al, 1994a) and nutritional resources to sustain the energy metabolism (Gerweck et al, 1993). Using an in vitro assay and a different tumour model, it was demonstrated that the energy status was stable during oxygen deprivation but with the availability of sufficient glucose (Gerweck et al, 1993). Moreover, the inhibition of glycolysis by 2-deoxyglucose and insulin caused a decrease in the ATP/$P_i$ ratio of an experimental sarcoma rat tumour model (Karczmar et al, 1992).

### Repetition time and assessment of bioenergetic status

The present study showed that a 6-s pulse interval (compared with 2 s) caused a higher signal intensity increase of $P_i$ than of β-NTP, which resulted in a reduction in the β-NTP/$P_i$ ratio of about 1.5, but there was no additional increase in the $P_i$ signal intensity after breathing oxygen 100% or carbogen. Therefore, the improvement in $P_i$ intensity by using $TR_{6s}$ is most probably because the $T_1$ effect of $P_i$ was minimized whereas the suggested paramagnetic effect of oxygen on $P_i$ was less important in this tumour model. $T_1$ of $P_i$ and β-NTP was not measured in the current study, but Okunieff (1988) reported $T_1$ values of 5.93 s for $P_i$ of anoxic tumours in mice that had been dead for 60 min. Moreover, the $T_1$ of phosphorus resonances was found to differ significantly between tumour models and to be dependent on tumour volume and on the oxygenation status of the tumour (Okunieff et al, 1986, 1987, 1988; Olsen et al, 1994, 1995). Finally, in vitro experiments have documented that metallic ions, probably present in the debris of necrotic regions are also likely to reduce $T_1$ of $P_i$ (McCain, 1987).

### Conclusion

In conclusion, inhalation of carbon monoxide was associated with enhanced radiation resistance, a decrease in tumour oxygenation and unchanged bioenergetic status expressed by the β-NTP/$P_i$ ratio. These results suggest that in this tumour model cells can remain metabolically active even at low oxygen tensions, which makes the ability of $^{31}$P-MRS to detect changes in low tumour $pO_2$ dependent on the potential of the tumour to produce high energy phosphates by anaerobic glycolysis. Induction of hyperoxia by breathing carbogen or oxygen (100%) was followed by an increase in β-NTP/$P_i$, tumour $pO_2$ and an enhancement of radiation response as a function of gas-breathing time.

### ACKNOWLEDGEMENTS

This study received financial support by Danish Cancer Society, Karen Elise Jensens Fond and Fru Agnes Niebuhr Anderssons Cancerforskningsfond. The authors would like to thank Ms M Andersen, Ms I M Johansen, Ms M Simonsen and Mr P Daugaard for excellent technical assistance.

### REFERENCES

Brizel D, Lin S, Johnson JL, Brooks J, Dewhirst MW and Piantadosi CA (1995) The mechanisms by which hyperbaric oxygen and carbogen improve tumour oxygenation. *Br J Cancer* **72**: 1120–1124

Brizel D, Scully SP, Harrelson JM, Layfield LJ, Bean JM, Prosnitz and Dewhirst MW (1996) Tumor oxygenation predicts for the likelihood of distant metastases in human soft tissue sarcoma. *Cancer Res* **56**: 941–943

Chaplin DJ, Horsman MR and Siemann DW (1993) Further evaluation of nicotinamide and carbogen as a strategy to reoxygenate hypoxic cells in vivo: importance of nicotinamide dose and pre-irradiation breathing time. *Br J Cancer* **68**: 269–273

Dixon WJ (1990) *BMDP Statistical Software Manual*, Vol. 2, pp. 1135–1155. University of California Press: Berkely, CA

Durand RE (1991) Keynote address: the influence of microenvironmental factors on the activity of radiation and drugs. *Int J Radiat Oncol Biol Phys* **20**: 253–258

Falk SJ, Ward R and Bleehen NM (1992) The influence of carbogen breathing on tumour tissue oxygenation in man evaluated by computerized pO2 histography. *Br J Cancer* **66**: 919–924

Gatenby RA, Kessler HB, Rosenblum JS et al (1988). Oxygen distribution in squamous cell carcinoma metastases and its relationship to outcome of radiation therapy. *Int J Radiat Oncol Biol Phys* **14**: 831–838

Gerweck LE, Seneviratne T and Gerweck KK (1993) Energy status and radiobiological hypoxia at specified oxygen concentrations. *Radiat Res* **135**: 69–74

Gerweck LE, Koutcher J and Zaidi ST (1995) Energy status parameters, hypoxia fraction and radiocurability across tumor types. *Acta Oncol* **34**: 335–338

Grau C and Overguaard J (1988) Effect of cancer chemotherapy on the hypoxic fraction of a solid tumor measured using a local tumor control assay. *Radiother Oncol* **13**: 301–309

Grau C, Horsman MR and Overgaard J (1992) Improving the radiation response in a C3H mouse mammary carcinoma by normobaric oxygen or carbogen breathing. *Int J Radiat Oncol Biol Phys* **22**: 415–419

Grau C, Khalil AA, Nordsmark M, Horsman MR and Overgaard J (1994) The relationship between carbon monoxide breathing, tumour oxygenation and local tumour control in the C3H mammary carcinoma in vivo. *Br J Cancer* **69**: 50–57

Horsman MR, Khalil AA, Nordsmark M, Grau C, Overgaard J (1993) Relationship between radiobiological hypoxia and direct estimates of tumour oxygenation in a mouse tumour model. *Radiother Oncol* **28**: 69–71

Horsman MR, Siemann DW, Nordsmark M, Khalil AA, Overgaard J and Chaplin DJ (1994) The combination of nicotinamide and carbogen breathing to improve tumour oxygenation prior to radiation treatment. *Adv Exp Med Biol* **361**: 635–642

Horsman MR, Khalil AA, Nordsmark M et al (1995) The use of oxygen electrodes to predict radiobiological hypoxia in tumors. In *Tumor Oxygenation*. Vaupel PW, Kelleher DK and Günderoth M (eds), pp. 49–57. Gustav Fischer: Stuttgart

Höckel M, Schlenger K, Aral B, Mitze M, Schäffer U and Vaupel P (1996) Association between tumor hypoxia and malignant progression in advanced cancer of the uterine cervix. *Cancer Res* **56**: 4509–4515

Kallinowski F, Zander R, Höckel, M and Vaupel P (1990) Tumour tissue oxygenation as evaluated by computerized-pO2-histography. *Int J Radiat Oncol Biol Phys* **19**: 953–961

Karczmar G, Arbeit JM, Toy BJ, Speder A and Weiner MW (1992) Selective depletion of tumor ATP by 2-deoxyglucose and insulin, detected by 31P magnetic resonance spectroscopy. *Cancer Res* **52**: 71–76

Kolstad P (1968) Vascularization, oxygen tension, and radiocurability in cancer of the cervix. *Norwegian Monographs on Medical Science*. Scandinavian University Books: Oslo

Lyng H, Olsen DR, Southon TE and Rofstad EK (1993) 31P-nuclear magnetic resonance spectroscopy in vivo of six human melanoma xenograft lines: tumour bioenergetic status and blood supply. *Br J Cancer* **68**: 1061–1070

McCain DC (1987) 31P nuclear spin relaxation. In *Phosphorus NMR in biology*. Burt CT (ed), pp. 25–61. CRC Press: Boca Raton, FL

Martin L, Lartigau E, Weeger P et al (1993) Changes in the oxygenation of head and neck tumors during carbogen breathing. *Radiother Oncol* **27**: 123–130

Moulder JE and Rockwell S (1984) Hypoxic fractions of solid tumors: experimental techniques, methods of analysis, and a survey of existing data. *Int J Radiat Oncol Biol Phys* **10**: 695–712

Nordsmark M, Grau C, Horsman MR, Jörgensen HS and Overgaard J (1995) Relationship between tumour oxygenation, bioenergetic status and radiobiological hypoxia in an experimental model. *Acta Oncol* **34**: 329–334

Nordsmark M, Overgaard M and Overgaard J (1996) Pretreatment oxygenation predicts radiation response in advanced squamous cell carcinoma of the head and neck. *Radiother Oncol* **41**: 31–39

Okunieff PG, Koutcher JA, Gerweck L, McFarland E, Hitzig B, Urano M, Brady T, Neuringer L and Suit HD (1986) Tumor size dependent changes in a murine fibrosarcoma: Use of in vivo 31P NMR for non-invasive evaluation of tumor metabolic status. *Int J Radiat Oncol Biol Phys* **12**: 793–799

Okunieff P, McFarland E, Rummeny E, Willett C, Hitzig B, Neuringer L and Suit H (1987) Effects of oxygen on the metabolism of murine tumors using in vivo phosphorus-31 NMR. *Amer J Clin Oncol* **10**: 475–482

Okunieff P, Ramsay J, Tokuhiro T, Hitzig B, Rummeny E, McFarland E, Neuringer LJ and Suit H (1988) Estimation of tumor oxygenation and metabolic rate using 31P MRS: Correlation of longitudinal relaxation with tumor growth rate and DNA synthesis. *Int J Radiat Oncol Biol Phys* **14**: 1185–1195

Olsen DR, Lyng H, Southon TE and Rofstad EK (1994) 31P-nuclear magnetic resonance spectroscopy in vivo of four human melanoma xenograft lines: spin-lattice relaxation times. *Radiother Oncol* **32**: 54–62

Olsen DR, Lyng H, Petersen S and Rofstad EK (1995) Spin-lattice relaxation time of inorganic phosphate in human tumor xenografts measured in vivo by 31P-magnetic resonance spectroscopy. Influence of oxygen tension. *Acta Oncol* **34**: 339–343

Overgaard J (1980) Effect of misonidazole and hyperthermia on the radiosensitivity of a C3H mouse mammary carcinoma and its surrounding normal tissue. *Br J Cancer* **41**: 10–21

© Cancer Research Campaign 1997

Overgaard J and Horsman MR (1996) Modification of hypoxia-induced radioresistance in tumors by the use of oxygen and sensitizers. *Sem Rad Oncol* **6**: 10–21

Raleigh JA, Dewhirst MW and Thrall DE (1996) Measuring tumor hypoxia. *Sem Rad Oncol* **6**: 37–45

Rofstad EK, Fenton BM and Sutherland RM (1988*a*) Intracapillary HbO2 saturations in murine tumours and human tumour xenografts measured by cryospectrophotometry: Relationship to tumour volume, tumour pH and fraction of radiobiologically hypoxic cells. *Br J Cancer* **57**: 494–502

Rofstad EK, DeMuth P, Fenton BM and Sutherland RM (1988*b*) 31P nuclear magnetic resonance spectroscopy studies of tumor energy metabolism and its relationship to intracapillary oxyhemoglobin saturation status and tumor hypoxia. *Cancer Res* **48**: 5440–5446

Rubin P, Hanley J, Keys HM, Marcial V and Brady L (1979) Carbogen breathing during radiation therapy. The radiation therapy oncology group study. *Int J Radiat Oncol Biol Phys* **5**: 1963–1970

Siemann DW, Hill RP and Bush RS (1977) The importance of the pre-irradiation breathing times of oxygen and carbogen (5% $CO_2$: 95% $O_2$) on the in vivo radiation response of a murine sarcoma. *Int J Radiat Oncol Biol Phys* **2**: 903–911

Sostman HD, Rockwell S, Sylvia AL et al (1991) Evaluation of BA1112 Rhabdomyosarcoma oxygenation with microelectrodes, optical spectrophotometry, radiosensitivity, and magnetic resonance spectroscopy. *Magnetic Reson Med* **20**: 253–267

Stone HB, Brown MJ, Phillips TL, Sutherland RM (1993) Oxygenation in Human Tumors: Correlation between Methods of Measurement and Response to Therapy. *Radiat Res* **136**: 422–434

Teicher BA, Lazo JS and Sartorelli AC (1981) Classification of antineoplastic agents by their selective toxicities towards oxygenated and hypoxic cells. *Cancer Res* **41**: 73–81

Van Den Boogart A, Howe FA, Rodrigues LM Stubbs M and Griffiths Jr (1995) In vivo 31P MRS: Absolute concentrations, signal-to-noise and prior knowledge. *NMR Biomed* **8**: 87–93

Van Der Veen JW, de Beer R, Luyten PR and van Ormondt D (1988) Accurate quantification of in vivo 31P NMR signals using the variable projection method and prior knowledge. *Magnetic Reson Med* **6**: 92–98

Vaupel P, Okunieff P, Kallinowski F and Neuringer LJ (1989). Correlations between 31P-NMR spectroscopy and tissue $O_2$ tension measurements in a murine fibrosarcoma. *Radiat Res* **120**: 477–493

Vaupel P, Okunieff P and Neuringer LJ (1990) In vivo 31P-NMR spectroscopy of murine tumours before and after localized hyperthermia. *Int J Hyperthermia* **6**: 15–31

Vaupel P, Kelleher DK and Engel T (1994*a*) Stable bioenergetic status despite substantial changes in blood flow and tissue oxygenation in a rat tumour. *Br J Cancer* **69**: 46–49

© Cancer Research Campaign 1997